# Widely Targeted Volatilomics and Metabolomics Analysis Reveal the Metabolic Composition and Diversity of Zingiberaceae Plants

**DOI:** 10.3390/metabo13060700

**Published:** 2023-05-27

**Authors:** Youjin Zhang, Rongxiu Su, Honglun Yuan, Haihong Zhou, Yiding Jiangfang, Xianqing Liu, Jie Luo

**Affiliations:** 1 Hainan Yazhou Bay Seed Laboratory, Sanya Nanfan Research Institute of Hainan University, Sanya 572025, China; 20090100210056@hainanu.edu.cn (Y.Z.); rongxiu.su@hainanu.edu.cn (R.S.); 183217@hainanu.edu.cn (H.Y.);; 2 College of Tropical Crops, Hainan University, Haikou 570288, China; 3 National Key Laboratory of Crop Genetic Improvement and National Center of Plant Gene Research (Wuhan), Huazhong Agricultural University, Wuhan 430070, China

**Keywords:** Zingiberaceae plants, widely targeted method, volatilome, metabolome, terpenoids, lipids

## Abstract

Zingiberaceae plants are widely used in the food and pharmaceutical industries; however, research on the chemical composition and interspecific differences in the metabolome and volatilome of Zingiberaceae plants is still limited. In this study, seven species of Zingiberaceae plants were selected, including *Curcuma longa* L., *Zingiber officinale* Rosc., *Alpinia officinarum* Hance, *Alpinia tonkinensis* Gagnep, *Amomum tsaoko* Crevost et Lemarie, *Alpinia hainanensis* K. Schum. and *Amomum villosum* Lour. *Myristica fragrans* Houtt. was also selected due to its flavor being similar to that of the Zingiberaceae plant. The metabolome and volatilome of selected plants were profiled by widely targeted approaches; 542 volatiles and 738 non-volatile metabolites were detected, and β-myrcene, α-phellandrene and α-cadinene were detected in all the selected plants, while chamigren, thymol, perilla, acetocinnamone and *cis*-α-bisabolene were exclusively detected in certain Zingiberaceae plants. Differential analysis showed that some terpenoids, such as cadalene, cadalene-1,3,5-triene, cadalene-1,3,8-triene and (*E*)-β-farnesene, and some lipids, including palmitic acid, linoleic acid and oleic acid were amongst the most varied compounds in Zingiberaceae plants. In conclusion, this study provided comprehensive metabolome and volatilome profiles for Zingiberaceae plants and revealed the metabolic differences between these plants. The results of this study could be used as a guide for the nutrition and flavor improvement of Zingiberaceae plants.

## 1. Introduction

Zingiberaceae is a group of monocotyledonous plants that are widely distributed in south and southeast Asia, comprising 53 genera and around 1300 species [1]. *Alpinia* is the largest and most widely distributed genus in the family, with a total of 230 species [2]. *Amomum* Roxb. is the second largest genus after *Alpinia*, containing approximately 180 species [3]. In addition, the *Zingiber* genus is also an important genus in the family of Zingiberaceae, including about 141 species [4]. Zingiberaceae plants are extensively used in the food industry [5,6], and their rhizomes are known for their characteristic aromas; the rhizome of *Curcuma longa* and *Etlingera elatior* are widely used as food flavoring ingredients in cooking [1,7]. Moreover, the seeds of some Zingiberaceae species can be used as spices, such as the seeds of *Amomum villosum*, which have a “grassy” odorant frequently used in meat dishes [8]. In addition, Zingiberaceae plants possess significant medicinal value and have been reported for their anticancer, antioxidative, anti-inflammatory, antiplatelet, anti-ulcer, anticonvulsive and analgesic effects [9]. Therefore, Zingiberaceae plants are also widely used in traditional medicine and the pharmaceutical industry [10]. The rhizomes of some Zingiberaceae plants are frequently used in traditional Chinese medicine: the rhizomes of *Curcuma longa* were used as a household remedy for sprains and swellings [3], and the rhizomes of *Zingiber officinale* have anti-inflammatory, antioxidant, analgesic and cardiovascular health promoting activities [11]. Moreover, many Zingiberaceae seeds are utilized to treat various ailments; the seeds of *Amomum tsaoko*, for instance, are commonly used to boost immunity and the treatment of bloating, vomiting and malaria [12].

The metabolites of Zingiberaceae plants determine their flavor and medical properties. For instance, gingerol is well-known to give Zingiberaceae plants a pungent taste, while volatiles such as geranial, eucalyptol, β-linalool and bornyl acetate contribute to the unique aroma of Zingiberaceae plants [13]. On the other hand, curcumin is the main compound in the rhizome of *Curcuma longa* and is anti-inflammatory, antioxidant, antimutagenic, antidiabetic, antibacterial, hepatoprotective, expectorant and anticancerous [14]. In addition, it was discovered that the curcuminoids isolated from *Zingiber cassumunar* Roxb could prevent the H_2_O_2_-induced decrease in cell viability of thymocytes and protect living cells suffering from H_2_O_2_-induced oxidative stress [15]. The leaves of *Etlingera elatior* were found to have a high content of antioxidative phytochemicals such as *p*-hydroxybenzoic acid, ferulic acid and syringic acid, which could reduce the risk of cancer, cardiovascular disease and many other diseases [16]. The diarylheptanoids from *Amomum muricarpum* seeds were reported to act as phytoestrogens, anti-tumor promoters, antiplatelet aggregation, antioxidant, anti-influenza and anti-inflammatory compounds [17]. Therefore, analyzing the metabolite composition and variations among different species of Zingiberaceae plants is of great significance for their applications and breeding improvement.

Currently, there have been several studies using metabolomics to analyze the metabolite composition and variations of Zingiberaceae plants. A recent study reported the volatilome profiling of ten species of Zingiberaceae; a total of 162 compounds were identified, and most of the identified volatiles were monoterpenes and sesquiterpenes, in which (*E*)-labda-8(17),12-diene-15,16-dial, n-hexadecanoic acid, 4-methoxy-6-phenethyl-2H-pyran-2-one and L-β-pinene were found in high concentrations [18]. The authors also noted that monoterpenes and sesquiterpenes were significantly varied among Zingiberaceae plants. Another study researched the chemical composition of the essential oils of four Zingiberaceae species [19]. A total of 87 chemical components were detected, and the major compounds were α-terpinyl acetate, β-turmerone, α-zingiberene and 1,8-cineol. Further analysis revealed that *Elettaria cardamomum* L. Maton rhizome contains the highest content of α-terpinyl acetate, which occurs in low concentrations in *Curcuma Longa*, *Zingiber Officinale* and *Alpinia Officinarum*. In addition, by using liquid chromatography hyphenated with high-resolution tandem mass spectrometry, researchers studied the metabolome of the seeds of *Aframomum melegueta* K. Schum [20]. In total, 25 diarylheptanoids, five gingerol derivatives and nine phenolic/organic acids were annotated, and the antimicrobial, antioxidant and enzyme inhibitory effects of these compounds were investigated as well. Asamenew et al. reported the profiling of phenolic compounds in *Zingiber officinale* and *Kaempferia parviflora* Wall. [21]. They found that gingerol-related phenolic acid was detected only in *Zingiber officinale*, while methoxyflavones were identified exclusively in *Kaempferia parviflora*. The major constituents among 18 phenolic acids detected from *Zingiber officinale* were 6-gingerol, 8-gingerol, 10-gingerol, 1-dehydro-6-gingerdione and diacetoxy-8-gingerdiol; 3,5,7,3′,4′-pentamethoxyflavone and 5,7,4′-trimethoxyflavone were confirmed as predominant constituents among 13 methoxyflavones from *Kaempferia parviflora*. Nonetheless, further analysis is still required to fully dissect the metabolite composition and variations among different species of Zingiberaceae plants.

At present, non-targeted metabolomics and volatilomics are the most commonly used methods to profile plants' metabolome and volatilome [22,23]. The non-targeted method focuses on the analysis of all the detectable metabolites in a sample, including chemical unknowns. However, the low sensitivity of non-targeted methods hinders their detection and annotation coverage [24]. Widely targeted metabolomics technology is an effective strategy to combine the advantages of high sensitivity that are conferred by targeted profiling and the wide coverage rate achieved by non-targeted analysis [25]. We previously developed a liquid chromatography-mass spectrometry (LC-MS)-based widely targeted metabolomics method and used it to quantify metabolites in crops and fruits, including those with low-intensity signals [26,27,28]. Additionally, our group developed a gas chromatography-mass spectrometry (GC-MS)-based volatilomics method to profile the volatilomes of different rice accessions [29]. Due to the increased sensitivity of widely targeted volatilome technology, the number of annotated volatiles increased from 43 to 132 in rice grain. Therefore, we could adopt widely targeted methods to obtain a more comprehensive metabolic profile of Zingiberaceae plants.

In this study, seven species of Zingiberaceae plants, including *Curcuma longa* (CL), *Zingiber officinale* (ZO), *Alpinia officinarum* (AO), *Alpinia tonkinensis* (ATG), *Amomum tsaoko* (AT), *Alpinia hainanensis* (AH), *Amomum villosum* (AV), and *Myristica fragrans* Houtt. (MF) were selected, and their metabolome and volatilome were profiled by widely targeted metabolomics and volatilomics methods. Although *Myristica fragrans* does not belong to the Zingiberaceae family, it was selected in this study because it is also rich in monoterpenes and sesquiterpenes and has a similar flavor to some Zingiberaceae plants [30]. Thanks to the high sensitivity, high detection and annotation coverage of the widely targeted methods, we obtained more comprehensive volatilome and metabolite profiles of Zingiberaceae plants. Moreover, we revealed the metabolic differences within the Zingiberaceae species and found differences in the accumulation of geraniol and geranial in different genera of Zingiberaceae, which provides a guide for the application of Zingiberaceae plants in the food and pharmaceutical industries.

## 2. Materials and Methods

### 2.1. Plant Material

To study the metabolites composition and difference in Zingiberaceae plants, we selected seven Zingiberaceae species, including rhizomes of *Curcuma longa* L. (CL), *Zingiber officinale* Rosc. (ZO) and *Alpinia officinarum* Hance (AO), and seeds of *Alpinia tonkinensis* Gagnep (ATG), *Amomum tsaoko* Crevost et Lemarie (AT), *Alpinia hainanensis* K. Schum. (AH) and *Amomum villosum* Lour (AV), as well as seeds of *Myristica fragrans* Houtt. (MF). These Zingiberaceae plants were purchased from local stores in Haikou.

### 2.2. Chemicals

The hexane was acquired from Fisher Scientific (FL, NJ, USA). Calcium chloride dihydrate, sodium chloride, pyridine and EDTA were obtained from Sinopharm Chemical Reagent Co., Ltd. (Shanghai, China). Chromatographic-grade acetonitrile, acetic acid and methanol were purchased from Merck (Darmstadt, Germany). N-alkanes (C8-C20) and all standards were purchased from Shanghai Aladdin Biochemical Technology Co., Ltd. (Shanghai, China) and Sigma-Aldrich (St. Louis, MO, USA).

### 2.3. Sample Preparation

Two biological replicates were collected for each species. The samples were ground into powder using a grinder machine (MM400, Retsch) with steel balls at 28 Hz for 1 min or more. For volatilome profiling, 0.35 g of the resulting powder was transferred into a 22 mL glass headspace vial, incubated for 10 min at 37 °C, and then 0.7 g of CaCl_2_·2H_2_O and 0.7 mL of a 100 mM EDTA-NaOH solution (pH 7.5) were added, gently mixed and sonicated for 5 min. Two repetitions were performed for each species. Samples were pre-heated for 10 min at 50 °C and extracted for 20 min at 50 °C. To perform metabolomics analysis, 0.05–0.1 g of sample powder was suspended in a 70% methanol-water solution in a ratio of 500–1000 mL. Next, the samples were extracted by ultrasonic wave for 10 min at 50 Hz for a total of three times [31]. At the end of each time, vortex vibration and mixing were required.

### 2.4. GC-MS Analysis

The volatilomes were profiled by gas chromatography (7890A GC, Agilent Technologies, Santa Clara, CA, USA) with an Agilent 7000D mass selective detector. An HP-5 MS capillary column (30 m × 0.25 mm i.d, 0.25 μm film thickness, Agilent Technologies) was used to separate compounds. The temperature program was as follows: the initial column temperature was 40 °C, held for 3 min, with a temperature increase of 2 °C/min temperature of 160 °C, and a temperature increase of 50 °C/min to a final temperature of 300 °C after reaching 160 °C, followed by a 3 min preservation at 300 °C. The injection temperature was 270 °C in splitless mode with a 0.75 mm i.d. inlet liner tube (Agilent Technologies, Santa Clara, CA, USA). The flow rate was He 1.0 mL/min (99.999%). Volatiles were first detected by full scan mode; then, these signals were converted to multiple reaction monitoring (MRM) transitions and integrated into the MS2T library, according to a previous report [29]. A fiber with a usage count of ~70 was used to perform volatilomics analysis to ensure method reproducibility.

### 2.5. LC-MS Analysis

Non-targeted metabolic profiling analyses were performed with Q Exactive Focus Orbitrap LC-MS/MS (Thermo Scientific, Waltham, MA, USA). Scanning mass ranged from *m/z* 100–1000 with an accumulation time of 0.1 s. The MRM mode with QTRAP 6500 + LC-MS/MS (Shimadzu, Kyoto, Japan) was used for targeted metabolome analyses. The detection window was set to 80 s, and the targeted scanning time was 1.5 s. The chromatographic column was a C18 column (Shim-pack GLSS C18, 1.9 UM, 2.1 × 100, Shimadzu). Mobile phase A was 0.04% acetic acid-water solution, and mobile phase B was 0.04% acetic acid-methanol solution. The qualitative and quantitative chromatographic conditions were consistent.

### 2.6. Qualitative and Quantitative Analysis of Metabolomics Data

In the volatilomics analysis, the C8-C20 alkane standard mix solution was measured using the same temperature program to calculate the retention index (RI). Signals were deconvoluted by MS-DIAL (Version 4.70) and identified by comparing the deconvoluted mass spectra and RI with those reported in the NIST library (Version 2.3) [32]. The peak areas were integrated using the Agilent MassHunter Quantitative Analysis software and manually adjusted. The metabolomics data were processed with compound discoverer (CD) 3.1 software to obtain the mass-to-charge ratio, retention time, and MS/MS2 information of all detected substances. Then, the detected signals were automatically matched through the internally established reference libraries of chemical standard entries of software to predict and identify the metabolite information. Commercially available standards were purchased and analyzed to confirm the identification results. The peak areas were integrated using the MultiQuant™ MD software and manually adjusted.

### 2.7. Statistical Data Analysis

The phylogeny analysis was conducted by the National Center for Biotechnology Information (NCBI) [33] and was drawn using iTol (Interactive Tree of Life, https://itol.embl.de/ (accessed on 3 March 2023)). The pie charts of metabolite types were drawn using Origin 2023 (http://www.uone-tech.cn/Origin.html (accessed on 12 April 2023)). Venn diagram and principal component analysis (PCA) were performed using Sanger Box (http://sangerbox.com/ (accessed on 15 April 2023)). TBtools was used to normalize the relative intensities of metabolites (transformed the intensity value by log_2_), draw the heatmap and perform the clustering analysis of metabolites [34]. The accumulation of geraniol, geranial, linalool and myrcene in the monoterpene pathway of Zingiberaceae plants was analyzed through the Kyoto Encyclopedia of Genes and Genomes (KEGG) [35].

## 3. Results

### 3.1. Phylogeny Analysis of Zingiberaceae Plants

**Figure 1:**
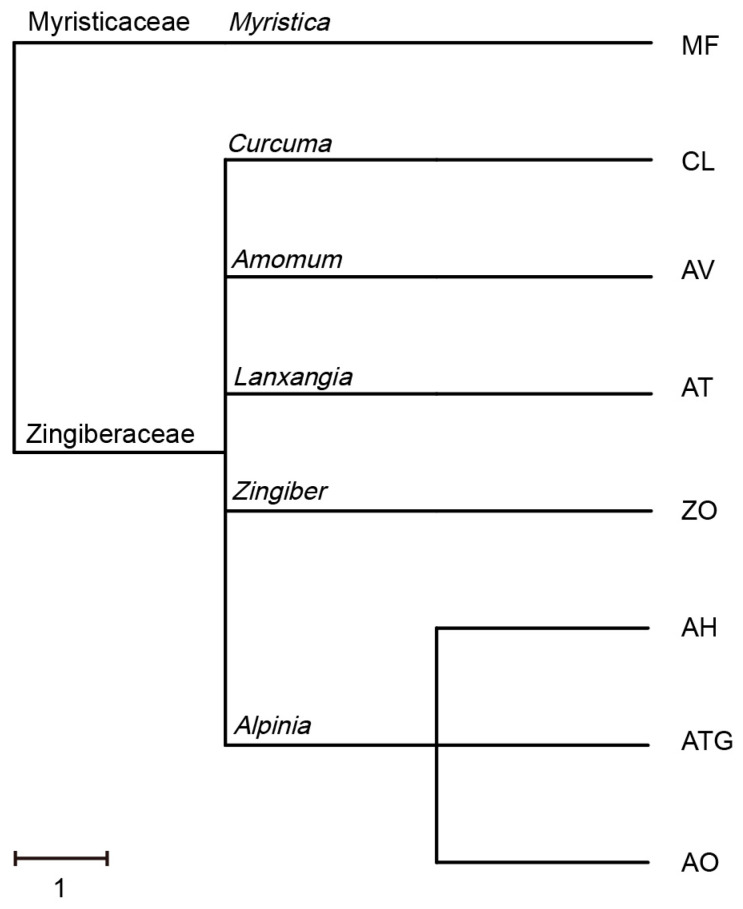
Phylogenetic tree analysis of seven species of Zingiberaceae plants.

The phylogeny analysis indicated that the selected Zingiberaceae plants belonged to five major genera: ATG, AH and AO belong to the *Alpinia* genus, while CL, AV, AT and ZO belong to the *Curcuma*, *AmomumL*, *Lanxangia* and *Zingiber* genera, respectively. In addition, MF belongs to the Myristicaceae *Myristica* genus (Figure 1). Next, a metabolomics analysis was conducted to investigate the metabolic profiles and differences in Zingiberaceae plants.

### 3.2. Volatilome and Metabolome Profiling of Zingiberaceae Plants

**Figure 2:**
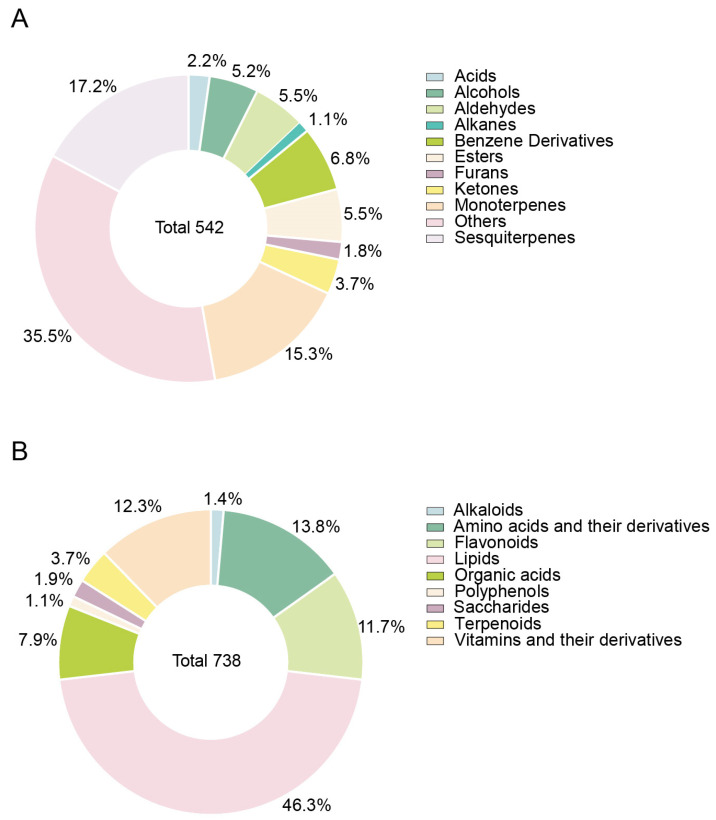
Classification of metabolome and volatilome data of Zingiberaceae plants. (**A**) Volatilome data. (**B**) Metabolome data.

The volatilome and metabolome of Zingiberaceae plants were profiled (Supplementary Tables S1 and S2). A total of 542 volatile compounds were detected, including 93 sesquiterpenes, 83 monoterpenes, 37 benzene derivatives, 30 esters, 30 aldehydes, 28 alcohols, 20 ketones, 12 acids, 10 furans, 6 alkanes and 193 other volatiles (Figure 2A). Additionally, 738 metabolites were detected, including 342 lipids, 102 amino acids and their derivatives, 91 vitamins and their derivatives, 86 flavonoids, 58 organic acids, 27 terpenoids, 14 sugars, 10 alkaloids and 8 polyphenols (Figure 2B). Overall, terpenoids and lipids are the main components of Zingiberaceae plants; zingiberene, eucalyptol, α-curcumene and γ-curcumen have the highest content in terpenoids, and the highest content of lipids compounds are LysoPC 16:0, LysoPC 18:1, LysoPC 18:2 and LysoPC 18:3.

### 3.3. Venn Diagram Analysis

**Figure 3:**
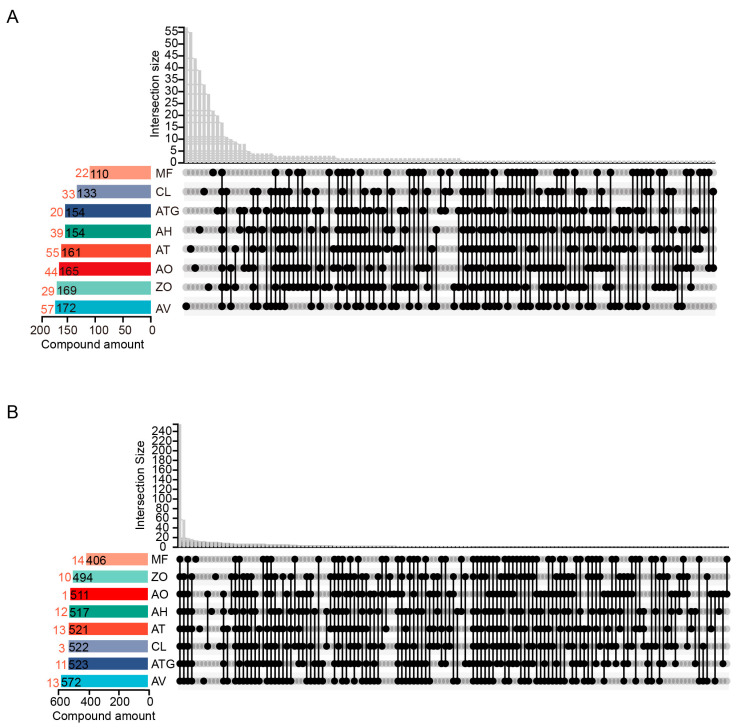
Venn diagram of metabolome and volatilome data of Zingiberaceae plants. (**A**) Volatilome data. (**B**) Metabolome data. Red number indicates the number of exclusive metabolites.

Based on the volatilome and metabolome profiles, we analyzed the metabolic similarities and differences of Zingiberaceae plants. Thirteen volatiles were detected in all selected Zingiberaceae plants, including β-myrcene, α-phellandrene and α-cadinene (Figure 3A). In addition, 57 volatiles, including chamigren, thymol and perilla, were exclusively found in AV, while 55 volatiles, including acetocinnamone and *cis*-α-bisabolene, were detected only in AT. Metabolomics analysis showed that 255 metabolites were detected in all selected Zingiberaceae plants, including LysoPC 18:3, LysoPC 18:1, stearic acid and palmitoleic acid (Figure 3B). Thirteen metabolites, such as 1-monopalmitin and 4-ketolutein, were exclusively measured in AT. In summary, AV and AT were found to have a greater diversity of volatiles and non-volatile metabolites in Zingiberaceae plants, respectively.

### 3.4. Principal Component Analysis

**Figure 4:**
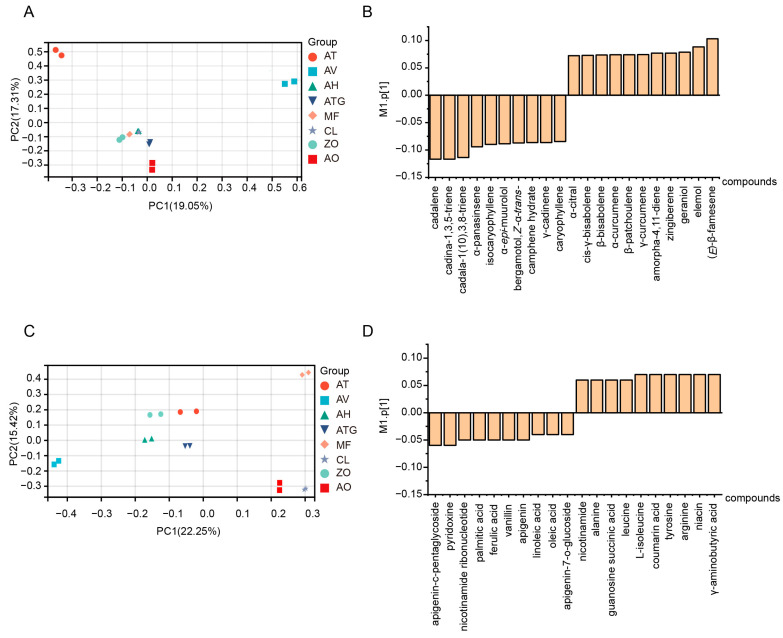
Principal component analysis (PCA) of metabolome and volatilome data of Zingiberaceae plants. (**A**) PCA plot of volatilome data. (**B**) Loading score of volatiles. (**C**) PCA plot of metabolome data. (**D**) Loading score of metabolites.

To obtain a deep insight into the metabolic composition and differences of Zingiberaceae plants, we conducted a principal component analysis (PCA). The first principal component (PC1) separated the Zingiberaceae plants in both plots, and PC1 accounted for 19.05% and 22.25% of the total variance in volatilome and metabolome data, respectively, which indicated that there might be significant metabolic differences among Zingiberaceae plants (Figure 4A,C). In addition, AV and AT were distinctly separated from other species in the PCA plot of volatilome data. AV, MF, CL and AO were distinctly separated from other species in the PCA plot of metabolome data, indicating the distinctively volatilome profile of AV and AT and the characteristic metabolome profile of AV, MF, CL and AO. Then, we calculated the loading scores to screen the differential metabolites in Zingiberaceae plants. The results revealed that in the volatilome data, cadalene, cadalene-1,3,5-triene, cadalene-1,3,8-triene, (*E*)-β-farnesene, elemol, geraniol and zingiberene have the highest absolute value of loading score, while in the metabolome data, γ-aminobutyric acid, L-isoleucine, niacin, arginine, pyridoxine, palmitic acid, linoleic acid and oleic acid have the highest absolute value of loading score (Figure 4B,D), which showed that these compounds might be responsible for the discrimination of Zingiberaceae plants.

### 3.5. Cluster Analysis

**Figure 5:**
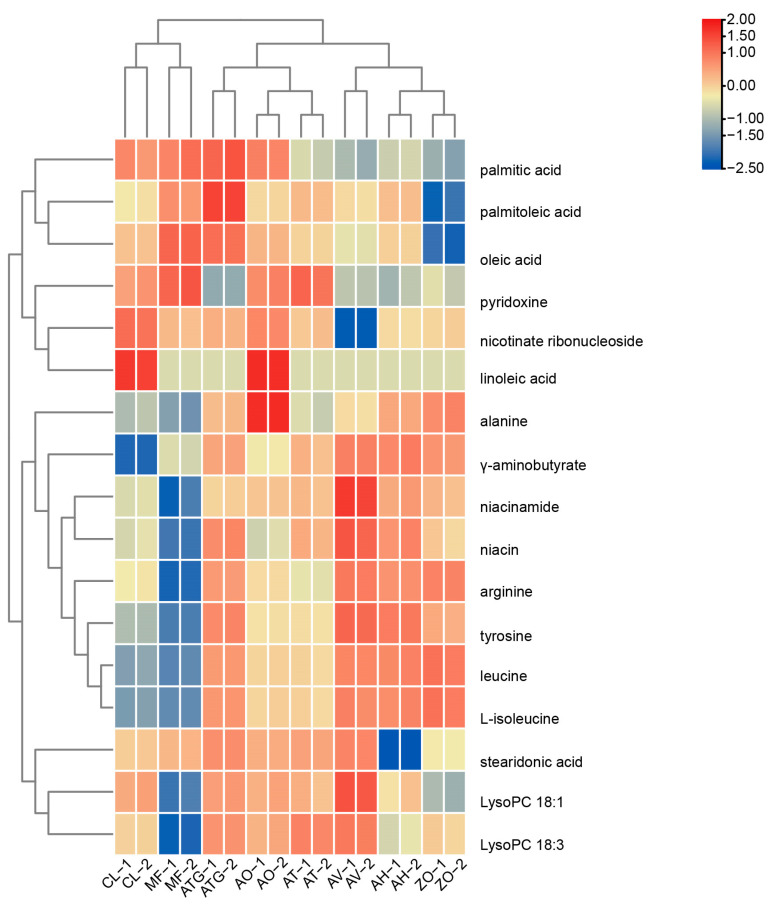
Heatmap obtained after hierarchical cluster analysis of amino acids, lipids and vitamins in Zingiberaceae plants. Two biological replicates were used for metabolomics analysis, expressed as 1 and 2, respectively. The relative intensity of metabolite was log2-transformed.

Venn diagram analysis and PCA revealed that amino acids, lipids, vitamins and terpenoids were significantly varied among Zingiberaceaes plants. Therefore, we conducted a cluster analysis on these metabolites (Figure 5). Based on the content of amino acids, lipids and vitamins, the selected Zingiberaceae plants can be divided into two clusters; the clustering of AV, AH and ZO was consistent with their distribution in PC1 of the metabolome PCA plot. A comparative analysis of metabolites in Zingiberaceae plants revealed that AV, AH and ZO were rich in amino acids, including arginine, leucine, L-isoleucine, tyrosine and γ-aminobutyrate. In addition, AV accumulated the most Lysopc 18:1, Lysopc 18:3, niacin and nicotinamide. ATG and CL were rich in lipids, such as oleic acid, palmitic acid, palmitoleic acid and stearic acid. AO produced more alanine and linoleic acid, and CL provided high content of linoleic acid and nicotinamide ribonucleotide.

**Figure 6:**
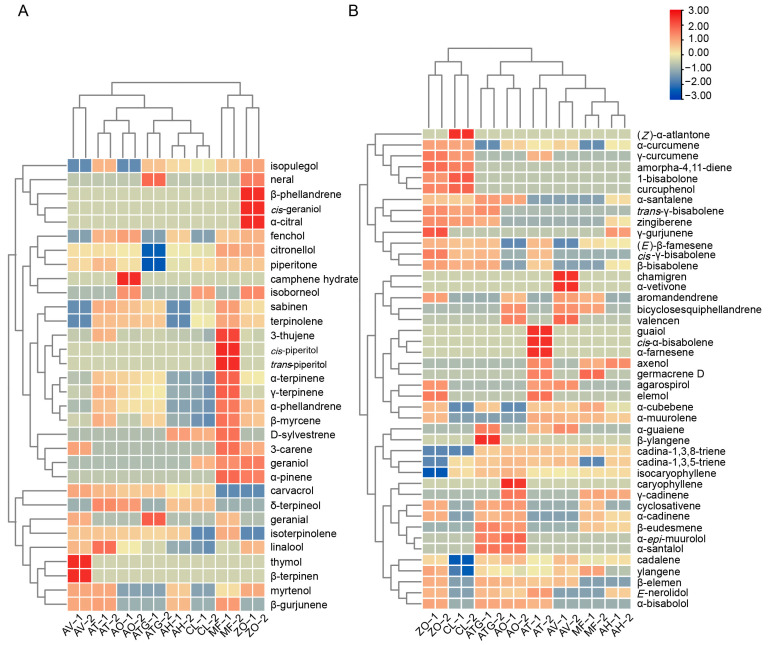
Heatmap obtained after hierarchical cluster analysis of monoterpenes and sesquiterpenes in Zingiberaceae plants. (**A**) Heatmap of monoterpenes. (**B**) Heatmap of sesquiterpenes. Two biological replicates were used for volatilomics analysis, expressed as 1 and 2, respectively. The relative intensity of metabolite was log_2_-transformed.

Clustering analysis of terpenoids in Zingiberaceae plants (Figure 6A,B) revealed that AV accumulated monoterpenes, including geranial, isoterpinolene, linalool, thymol, β-terpinen, myrtenol and β-gurjunene, and sesquiterpenes, such as chamigren and α-vetivone. AT was rich in α-terpinene, γ-terpinene, α-phellandrene, β-myrcene and other monoterpenes, as well as guaiol, *cis*-α-bisabolene, α-farnesene, axenol and some other sesquiterpenes. AO produced more monoterpenes, such as camphene hydrate and isoborneol, and more sesquiterpenes, including caryophyllene, γ-cadinene, cyclosativene, α-cadinene, β-eudesmene and α-*epi*-muurolol. ATG provided a high content of geranial, cadalene, ylangene, β-elemen, *E*-nerolidol and α-bisabolol. ZO has a high content of isopulegol, neral, β-phellandrene, *cis*-geraniol, α-citral, (*Z*)-α-atlantone, α-curcumene, γ-curcumene, amorpha-4,11-diene, 1-bisabolone and curcuphenol.

## 4. Discussion

The widely targeted methods enable us to obtain more comprehensive metabolome and volatilome profiles of Zingiberaceae plants. Zingiberaceae plants contain many complex volatiles, some of which are still unknown. In recent years, based on the non-targeted volatilomics approaches, a total of 58 secondary compounds were tentatively identified in four Malaysian *Zingiber officinale* Roscoe, and 130 metabolites were detected from seven common Zingiberaceae plants [36]. In this study, by integrating MS2 spectral tag library, we not only improved the method sensitivity but also broadened the annotation coverage and detected and annotated more compounds compared to previous reports in Zingiberaceae plants. In a similar study, researchers conducted a comparative metabolomic analysis of cordyceps by widely targeted metabolomics and non-targeted metabolomics; 778 and 1449 metabolites were identified by the non-targeted metabolomics and widely targeted metabolomics, respectively, which also demonstrated an increased annotation coverage of the widely targeted approach [37]. Overall, the widely targeted metabolomics method is more efficient than methods previously used and may considerably promote the study of plant metabolomes [38].

**Figure 7:**
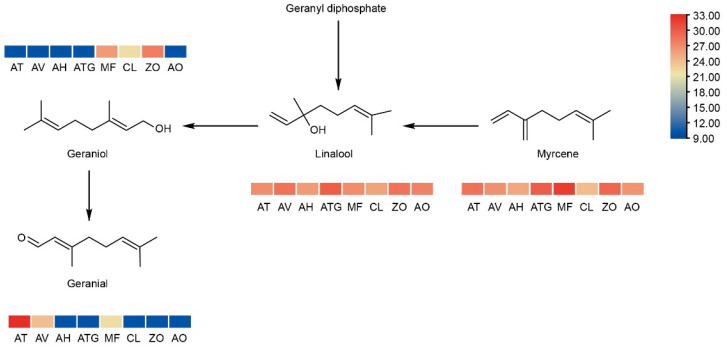
The synthetic pathway of geraniol and geranial. The relative intensity of metabolite was log_2_-transformed.

Monoterpenes such as linalool, geranial, geraniol and myrcene are known not only for their flavor contributions but also for their diverse biological activities [39,40,41]. Geraniol, linalool and myrcene were all generated with geranyl diphosphate as their precursor (Figure 7). Linalool is synthesized by linalool synthase using geranyl diphosphate as a precursor or by linalool dehydratase catalyzed myrcene [42], and myrcene is produced from geranyl diphosphate by myrcene synthase [43]. The results showed that linalool and myrcene are detected in all selected Zingiberaceae plants, indicating the similarity in this pathway of the selected Zingiberaceae species. In addition, geraniol is produced under the action of geranyl diphosphate diphosphatase and monoterpene diphosphatase; it can also be produced by linalool under the action of geraniol isomerase [44]. Geraniol exists in CL, ZO and MF, and geranial is the downstream product of geraniol; it is catalyzed by geraniol dehydrogenase. Geranial also exists in AV, AT and MF. In summary, geraniol and its downstream product, geranial, were not detected in *Alpinia* plants. The differences in the accumulation of geraniol and geranial in Zingiberaceae plants may be caused by the differences in gene expression and metabolic enzyme activities.

Zingiberaceae is a taxonomically complex family [45]. A recent report proposed an approach for comparing the metabolite content of plants and classifying plants by their metabolite content [46]. Accordingly, the metabolic differences can reflect their phylogenetic relationships to some extent. In previous studies, AT and AV are classified into the same genus [47]. However, a recent report considered that AT belonged to the *Lanxangia* genus [48]. In this study, the obvious separation of AV and AT in the volatilome PCA plot supports this point of view. In order to provide a metabolome-based classification of Zingiberaceae plants, marker metabolites are widely used to distinguish species. Researchers studied the chemical components of Zingiberaceae plants through a volatilomics analysis, and bornyl acetate, α-cadinol, linalool, β-myrcene, camphor, limonene, terpinolene and borneol were selected as the potential markers for discriminating AV and *Amomum villosum* Lour. var. xanthioides T. L. Wu et Senjen [3], and the major compounds found in ATG, CL, ZO and AO were α-terpinyl acetate, β-turmerone, α-zingiberene and 1,8-cineol, respectively [20]. In this study, we screened some potential markers to discriminate MF from Zingiberaceae plants; the content of these metabolites in MF is much higher than other selected Zingiberaceae plants, including 3-thujene, *cis*-piperitol, *trans*-piperitol and α-terpinene.

Bioactive compounds and nutritional compositions are differentially accumulated in Zingiberaceae plants. Our findings showed that compared to other Zingiberaceae plants, ZO contains the most abundant zingiberene and α-pinene. Zingiberene, a non-zinc-dependent inhibitor of histone deacetylase inhibitors class I, was applied in trauma-related neuropathic pain forms [49], and α-pinene has anti-inflammatory and wound healing activity [50]. ATG provided high content of α-bisabolol, and many studies demonstrated the pharmacological properties of α-bisabolol, including anticancer, antinociceptive, neuroprotective, cardioprotective and antimicrobial [51]. AT had high levels of α-phellandrene, and α-phellandrene was reported to have antinociceptive and antineoplastic properties [52]. AO produced more isoborneol and caryophyllene than other Zingiberaceae plants in our study, and isoborneol could be used as a natural and safe antisporulating agent for commercial applications to control spore infections of *Aspergillus flavus*; caryophyllene possesses significant anticancer activities, affecting growth and proliferation of numerous cancer cells [53,54]. In addition, we found AH and ZO were rich in amino acids; these amino acids are fundamental building blocks supporting life, such as arginine, which is involved in the biosynthesis of proteins, the host immune response, urea cycle and nitric oxide production [55]. Zingiberaceae plants have wide biological activities, and more laboratory investigations and product developments are needed.

## 5. Conclusions

This work conducted widely targeted volatilomics and metabolomics analyses of seven Zingiberaceae plants and *Myristica fragrans* Houtt., and detected 542 volatiles and 738 non-volatile metabolites. Further analysis revealed the most varied volatiles and metabolites, found differences in the accumulation of geraniol and geranial in different genera of Zingiberaceae, and screened some potential markers to discriminate *Myristica fragrans* Houtt. from Zingiberaceae plants. Specifically, *Amomum villosum* Lour was observed to have high concentrations of various amino acids, and *Alpinia tonkinensis* Gagnep and *Curcuma longa* L. were rich in most lipids, while *Zingiber officinale* Rosc. contained high contents of terpenoids, geraniol and its downstream product, geranial, were not detected in *Alpinia* plants. The potential markers, which may discriminate *Myristica fragrans* Houtt. from Zingiberaceae plants are 3-thujene, *cis*-piperitol, *trans*-piperitol and α-terpinene. Overall, this research provides new insight into the chemical composition and metabolic diversity of Zingiberaceae plants, which could be used as a guide for Zingiberaceae plants' application and breeding improvement.

## Data Availability

No new data were created or analyzed in this study. Data sharing is not applicable to this article.

## Supplementary Materials

The following supporting information can be downloaded at: https://www.mdpi.com/article/10.3390/metabo13060700/s1, Table S1: Metabolic signals in Zingiberaceae plants detected by GC-MS-based targeted; Table S2: Metabolic signals in Zingiberaceae plants detected by LC-MS-based targeted.
